# Morphological Characterization and Complete Mitochondrial Genome of a New Epizoic Hexamerous Brittle Star *Gymnolophus sexradia* sp. Nov

**DOI:** 10.1002/ece3.73841

**Published:** 2026-07-28

**Authors:** Ziye Zhang, Weilin Xiao, Chao Zhang, Chunyan Li, Huixian Zhang, Zelin Chen

**Affiliations:** ^1^ CAS Key Laboratory of Tropical Marine Bio‐Resources and Ecology (LMB), Guangdong Provincial Key Laboratory of Applied Marine Biology, South China Sea Institute of Oceanology, Chinese Academy of Sciences Guangzhou China; ^2^ University of Chinese Academy of Sciences Beijing China

**Keywords:** brittle star, epizoic, *Gymnolophus sexradia* sp. nov, micro‐CT, mitogenome, phylogeny, symbiosis

## Abstract

The brittle star of genus *Gymnolophus* Brock, 1888 (Ophiuroidea: Ophiotrichidae Ljungman, 1867) is commonly found in epizoic relationships with crinoid hosts, such as *Comaster schlegelii* (Crinoidea: Comatulidae Fleming, 1828), benefiting from food and protection. Here we describe a new hexamerous epizoic species, *Gymnolophus sexradia* sp. nov., from the South China Sea. We provide a detailed description of the ossicle structure, based on micro‐CT scanning, and sequences of 1464 nuclear exons and the complete mitogenome of the new species. The specimen features a hexamerous body plan and six arms which may indicate fissiparity. The shape and articulation of the arm vertebrae, along with the lateral arm plates and overlapping split dorsal arm plates, enhance arm flexibility in the horizontal direction, facilitating its grasping behavior toward the crinoid host. Phylogenetic analysis of nuclear exons clusters the new species with *Ophiomaza cacaotica* (Lyman, 1871) and *Gymnolophus obscura* (Ljungman, 1867). The complete mitogenome consists of 15,669 bp and 37 genes with a conserved gene order identical to that of *Ophiactis savignyi* and other studied mitogenomes in Ophiotrichidae. Notably, 14 residues in four mitochondrial genes were identified as positively selected, which may reflect adaptations linked to the epizoic lifestyle with crinoid hosts.

## Graphical abstract

This study describes a new hexamerous epizoic brittle star species, *Gymnolophus sexradia* sp. nov., from the South China Sea, which lives on crinoid hosts and exhibits a six‐armed body plan possibly indicating fissiparity. Its vertebral and arm plate structures enhance horizontal flexibility for grasping the host, and phylogenetic analysis based on nuclear exons and the complete mitogenome places it near *Gymnolophus obscura* and *Ophiomaza cacaotica* . Notably, 14 positively selected residues in four mitochondrial genes may reflect adaptations to its epizoic lifestyle on crinoids.

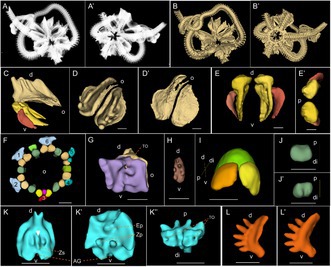

## 1 Introduction

Brittle Stars (Class: Ophiuroidea Gray, 1840) represent the most species‐rich group within the extant Echinodermata, exhibiting a wide distribution across various marine environments. Typically small, with a disc diameter ranging from 5 mm to 30 mm, they inhabit diverse substrates, from shallow coral reefs to the deep sea (Chen et al. 2022; Stöhr et al. 2012; Yulin and Ning 2012). There are 67 epizoic species listed with a specific taxonomic group of hosts by Stöhr out of 2136 ophiuroid species (Stöhr 2025), for example, *Ophiothrix lineata* resides within the sponge *Callyspongia vaginalis* (Lamarck, 1814), where it benefits from food and shelter while enhancing the sponge's pumping efficiency through its feeding and cleaning behaviors (Hendler et al. 1999). Epizoic species show distinct morphological characters from nonepizoic ones in body size, arm structure (Goharimanesh et al. 2022) and shape of spines (Kroh and Thuy 2013; Hendler et al. 1999). The larvae of *Amphipholis conolampadis*, a species that is symbiotic exclusively with the irregular sea urchin *Conolampas diomedeae*, possess hooked dorsal and ventral spines at the end of their arms, which disappear during the adult stage (Kroh and Thuy 2013), reflecting their adaptation to the host's microenvironment. The arms of brittle stars are more flexible than those of starfish, show a wide range of motion, important for feeding and locomotion. Epizoic ophiuroids are expected to curl their arms and grasp their host, developing distinct articular structures between arm vertebrae like the dorsal keeled vertebra in Ophiotrichidae (Goharimanesh et al. 2022).

Ophiotrichidae Ljungman, 1867 is a large family including 13 genera and 168 species (from WoRMS), but there is only one accepted species in *Gymnolophus* Brock 1888 and five in Ophiomaza Lyman, 1871, both consisting of epizoic species found on host Crinoidea. *Gymnolophus obscura* (Ljungman, 1967) (Family: Ophiotrichidae; Genus: *Gymnolophus*) primarily inhabits subtidal coral reef ecosystems, with its distribution ranging from Sri Lanka, the Philippines, Java, Ambon, to northern Australia, characterized by a bluish‐black body, large radial shields with a crest and sharp smooth spines (Nigam and Raghunathan 2016). *G. obscura* and *Ophiomaza cacotica* typically curl their arms around the central disc of host crinoids. *G. obscura* feeds on material filtered by *C. schlegelii* , one of their preferred hosts (Mekhova et al. 2018), and its diet mirrors that of its host (Li et al. 2023). Additionally, through host switching, *G. obscura* avoids intraspecific competition and gains access to more favorable nutrient sources and protective conditions (Mekhova et al. 2018). It may also exploit the chemical defense mechanisms of *C. schlegelii* to mitigate predation by reef fish (Kasumyan et al. 2020).

The epizoic lifestyle of animals may simultaneously drive mitochondrial genome evolution, morphological specialization, and redistribution of energy metabolism. In Seisonidea, the mitochondrial gene arrangement supports its close relationship with the endoparasitic acanthocephala as an epizoic group, suggesting that epibiosis may be a transitional stage from free‐living to parasitic life (Sielaff et al. 2016). Its body morphology also exhibits host‐dependent characteristics, such as the absence of typical rotifer wheel structures and only small ciliary brushes at the mouth; further genomic and transcriptomic studies show that with increasing host dependence, Seisonidea may undergo gradual gene loss and mitochondrial haplotype differentiation (Mauer et al. 2021). Similarly, the whale barnacle enhances its attachment and resistance to water flow through a special shell base and short, thick cirri, reflecting the morphological adaptation of epizoic animals to the host's surface environment (Kim et al. 2020). Therefore, changes in the mitochondrial genome of epizoic animals may reflect their phylogenetic and host‐dependent evolution, while morphological specialization helps them reduce movement costs and enhance attachment; correspondingly, energy metabolism may be redistributed between reducing free‐living consumption and responding to host surface water flow, attachment, and stress pressure.

In this study, we present the first detailed analysis of the skeleton morphology and complete mitogenome of a new hexamerous species *Gymnolophus sexradia* sp. nov. collected from the South China Sea. We compare these findings with mitochondrial genomes and nuclear exons from other species within the family Ophiotrichidae. The arm ossicle shape and articular structure support significant flexibility in both ventral and horizontal directions, which may represent an adaptive evolution that aids in grasping the host crinoid. We also reveal the mitochondrial genome features of *G. sexradia* and place it between *Ophiomaza cacotica* and *Gymnolophus obscura* within the family Ophiotrichidae, and identify positive selective signals at 14 codons in the ATP6, COIII, ND4, and ND5 genes. This study provides valuable morphological and molecular resources for future research on the co‐evolution of *Gymnolophus* and their crinoid hosts.

## 2 Materials and Methods

### 2.1 Sampling and External Observation

Specimens of the new species were collected near the Nansha Islands in the South China Sea by divers in July 2022 and stored in 75% ethanol on board. Specimens were examined and photographed using a Nikon D800 camera (whole body) and a stereoscopic microscope (Zeiss Axio Observer). Skin on the ossicle surface was dissolved using a commercial bleach (Tianli). All measurements were performed using ImageJ based on the photographs of specimens or using 3D Slicer based on micro‐CT datasets. Since the species does not fall under endangered or protected categories, no special collection license was required for this study. The following resources were consulted when describing the morphology: the WoRMS database (https://www.marinespecies.org/), (Brock 1888; Nigam and Raghunathan 2016; Mekhova et al. 2018; Li et al. 2023).

### 2.2 Micro‐CT Scanning and Ossicle Segmentation

The specimen was scanned using a Hiscan XM Micro‐CT scanner (Suzhou Hiscan Information Technology Co. Ltd). The X‐ray tube was set to 80 kV and 100 μA, and images were captured at a resolution of 35 μm according to the body size. A 0.5° rotation step through a 360° angular range with a 50 ms exposure per step was employed. The images were reconstructed using Hiscan Reconstruct software (Version 3.0, Suzhou Hiscan Information Technology Co. Ltd) and analyzed with Hiscan Analyzer software (Version 3.0, Suzhou Hiscan Information Technology Co. Ltd). DICOM files were imported and visualized in 3D Slicer. Ossicle segmentation was performed semi‐manually using the “Fill between slices” tool in the “Segment Editor” module. In detail, we chose one slice, painted the region of the target ossicle using the “paint” tool with a CT value greater than 2100, then moved to another slice in steps of 2 to 5 slices depending on the variation between slices, and repeated this process until the entire ossicle of interest was covered. We then filled all gaps between the painted slices using the “Fill between slices” tool to obtain the final segment. The CT values of the segmented ossicles were analyzed using the “Segment Statistics” module and converted to stereom density (*ρ*) using the following formula, derived from a hydroxyapatite (HA) calibration phantom: *ρ* = 0.9039737 + 0.0001754 * CT.

### 2.3 Sequence Assembly and Mitochondrial Gene Prediction

Total genomic DNA of the specimen was extracted using the TIANamp Marine Animal DNA Kit (Tiangen Biotechnology Co. Ltd., Beijing, China) following the manufacturer's standard operating protocol. Extracted DNA was sequenced on the DNBSEQ‐T7 platform (Mgi Tech Co. Ltd). Paired‐end reads of length 150 bp were cleaned using Trimmomatic (ver. 0.39) with the following parameters: “PE–threads 8–phred33 ILLUMINACLIP:$adapt:2:30:10:8:true LEADING:3 SLIDINGWINDOW:20:20 MINLEN:40”. A total of 325 million cleaned read pairs were assembled using NOVOPlasty v4.3.1 (Dierckxsens et al. 2017) with an expected genome size range of 15,000 bp–18,000 bp and a k‐mer size of 35, using all complete ophiuroid mitogenomes obtained from NCBI as seed sequences. The NOVOPlasty assembly contained two overlapping circularized contigs, which were joined manually at the overlap region and then shifted to place the start codon of COI at position 1 (1‐based) in the final genomic FASTA file. Genes of the assembled mitogenome were predicted using MITOS2 (https://mitos.bioinf.uni‐leipzig.de/index.py, accessed on 12 April 2024) (Donath et al. 2019). Nucleotide sequences of all coding genes were aligned to those of closely related species obtained from publicly available databases and reviewed to ensure the completeness and accuracy of the sequences. A de novo assembly of 2081 Mbp was also obtained using Megahit assembler with the following parameters: –min‐count 2 –bubble‐level 2–m 100,000,000,000–t 64 –min‐contig‐len 400. A single contig (k141_2821769) of size 15,810 bp was identified by BLASTN against public ophiuroid mitogenomes. The last 141 bp of the contig overlapped the head exactly, indicating a circularized contig. Therefore, it was trimmed to 15,669 bp after circularization. The final de novo mitogenome was almost identical to the NOVOPlasty assembly (only 1 bp mismatch).

### 2.4 Phylogenetic Analysis

To investigate the mitogenome characteristics within the family Ophiotrichidae, we included the mitogenome sequences of eight ophiuroid species from the NCBI database: *Ophiothrix* sp. (NC_082173), *Ophiothrix proteus* (OM967070), *Ophiothrix* sp. (ON457157), *Ophiothrix exigua* (NC_082173), *Macrophiothrix* sp. (OM994402), *Macrophiothrix* sp. (ON758773), *Ophiothrix scotiosa* (ON051653), and *Macrophiothrix demessa* (OM970880). *Ophiactis savignyi* (NC_065087) was used as an outgroup. Amino acid sequences of each protein‐coding gene (PCG) were aligned using MAFFT v7.475 with the parameters: –ep 0.2 –maxiterate 1000 –localpair, and then transformed back to nucleotide multiple sequence alignment (MSA). Conserved regions of each MSA were retained using GBlocks v0.91b (Castresana 2000) with default settings. All MSAs of the 13 PCGs were concatenated (gene by gene) to generate a final FASTA file including nine nucleotide sequences (one per species) for phylogenetic analysis. The top two best‐fit models (highest AIC or BIC value), GTR + G + I and GTR + G, were selected by MEGA's ModelTest (Hall 2013). Maximum likelihood phylogenetic reconstruction was conducted with RAxML v8.2.12 (Stamatakis 2014) using the PROTGAMMAAUTO model for amino acid sequences and the GTR + G + I (or GTR + G) substitution model for nucleotide sequences, and rapid bootstrap analysis (‐N 1000) for the concatenated PCGs of the nine mitogenomes. Data visualization was carried out using the ggplot2 package in R v4.3.1. The two models generated the same phylogenetic topology. COI nucleotide sequences of Ophiotrichidae longer than 600 bp were downloaded from NCBI. The Kimura distance was computed using dnadist and the Kimura 1980 (K80) model in PHYLIP (version 3.697) (Mansour 2009) (see results in Table S3).

Exon nucleotide sequences of 576 ophiuroid species were obtained from (O'Hara et al. 2017). Each exon sequence was aligned to our MEGAHIT assembly using NCBI tblastn, and the best‐hit region along with its corresponding sequence was retrieved. MSAs were performed using MAFFT v7.505 with the following parameters: –localpair –addfull Gym.exon.fa other.exon.fa, where Gym.exon.fa is the FASTA file containing the exon sequences of the new species and other.exon.fa contains sequences from Ophiotrichidae and *Ophiopholis* Müller & Troschel, 1842 (as outgroup). All MSAs with aligned exon sequences were concatenated (exon by exon) to generate a final FASTA file for phylogenetic analysis. Maximum likelihood phylogenetic reconstruction was conducted with RAxML v8.2.12 (Stamatakis 2014) using the same model as in (O'Hara et al. 2017), that is, 200 GTR + CAT rapid bootstrap trees followed by a full GTR + GAMMA maximum likelihood search.

### 2.5 Prediction and Visualization of tRNA Secondary Structures

The secondary structures of the 22 tRNAs were predicted using the RNAfold tool from the ViennaRNA package (version 2.5.0) (Varenyk et al. 2023), with the options “‐d2 –noLP –temp = 25 –MEA–p2”. Structures were reviewed, and constraint files were manually created for the second run of RNAfold to predict the cloverleaf structure. The final secondary structures were visualized using the “relplot.pl.” script from the same package.

### 2.6 Nucleotide Diversity and Selection Pressure Analysis

Positive selection analyses were performed using “codeml” from PAML v4.9j (Yang 2007) and the MSA of mitochondrial PCGs. The maximum likelihood (ML) tree was used as the tree topology. Branch models with one‐ratio, two‐ratio (with *Gymnolophus sexradia* sp. nov. as the foreground), and free‐ratio were employed for the mitogenome analysis. The branch‐site model was used to identify genes under positive selection in the foreground lineage. Bayes Empirical Bayes (BEB) analysis was used to calculate the Bayesian posterior probabilities of positively selected sites (posterior probability > 0.9).

## 3 Results

### 3.1 External Observations

**FIGURE 1:**
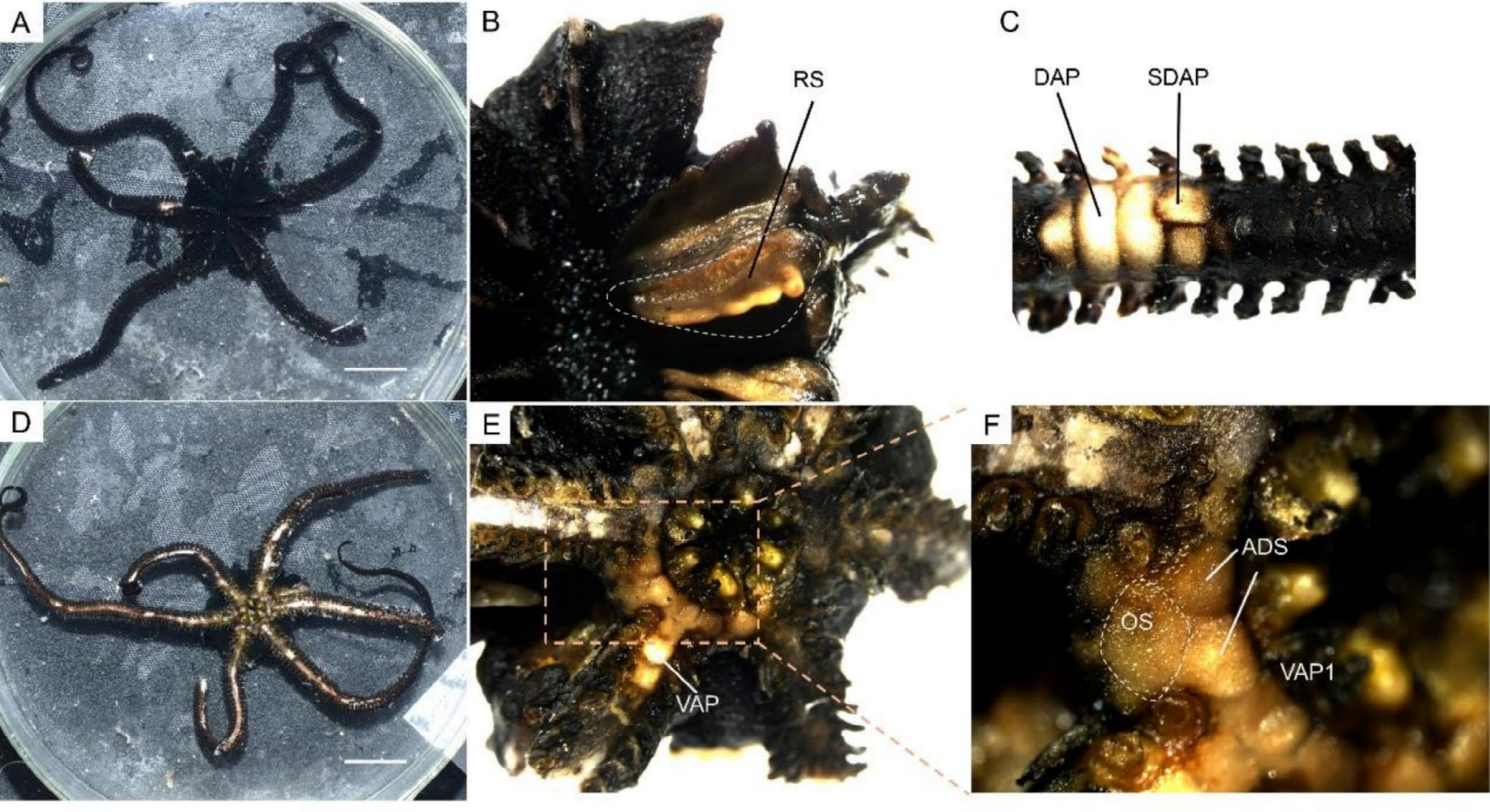
External morphological observation of *Gymnolophus sexradia* sp. nov. (A) Dorsal view of brittle star, scale bar: 1 cm; (B) Skinned and naked radial shields; (C) Skinned and naked dorsal arm plates; (D) Ventral view of brittle star, scale bar: 1 cm; (E) Mouth and ventral arm plates; (F) Detail of oral shield, adoral shield and the first ventral arm plate. RS, radial shield; DAP, dorsal arm plate; SDAP, split dorsal arm plate; VAP, ventral arm plate; VAP1, first ventral arm plate; OS, oral shield; ADS, adoral shield.

The specimen, collected with the host crinoid from the South China Sea, is large with a disc diameter of 2.0 cm. The entire body plan is hexamerous. The dorsal disc is covered by thick black skin bearing scattered granules, and lacks a distinct central scale. The skin is thinner and the color is lighter (brown to yellow white) on the ventral side (Figure 1A,D). The radial shields are large and elongated, measuring more than four‐fifths of the disc radius. They are triangular in shape and do not contact each other, but the space between them is tiny. Each radial shield bears a tall, prominent radial crest that decreases in size from the disc edge toward the center (Figure 1B). The oral shield is small, diamond‐shaped with rounded angles, wider than it is long. The adoral shields are smaller than the oral shields, rounded pentagons that connect to each other and overlap with the adjacent oral shield. Six pairs of adoral shields and six first‐ventral arm plates form a ring around the mouth with six jaws (Figure 1E,F). The specimen possesses six long arms, each covered with black skin and rolled up at the tip, more flexible horizontally than vertically. The dorsal arm plate is rectangular to spindle‐shaped, strongly arched and almost keeled, approximately three times as wide as it is long (Figure 1C). The ventral arm plate is nearly rectangular, slightly convex distally. The lateral arm plates articulate with two to four spines in the first six to seven segments, increasing to five spines in the subsequent segments, and then decreasing to one or two spines at the tip segments.

### 3.2 Micro CT Skeleton Characters

**FIGURE 2:**
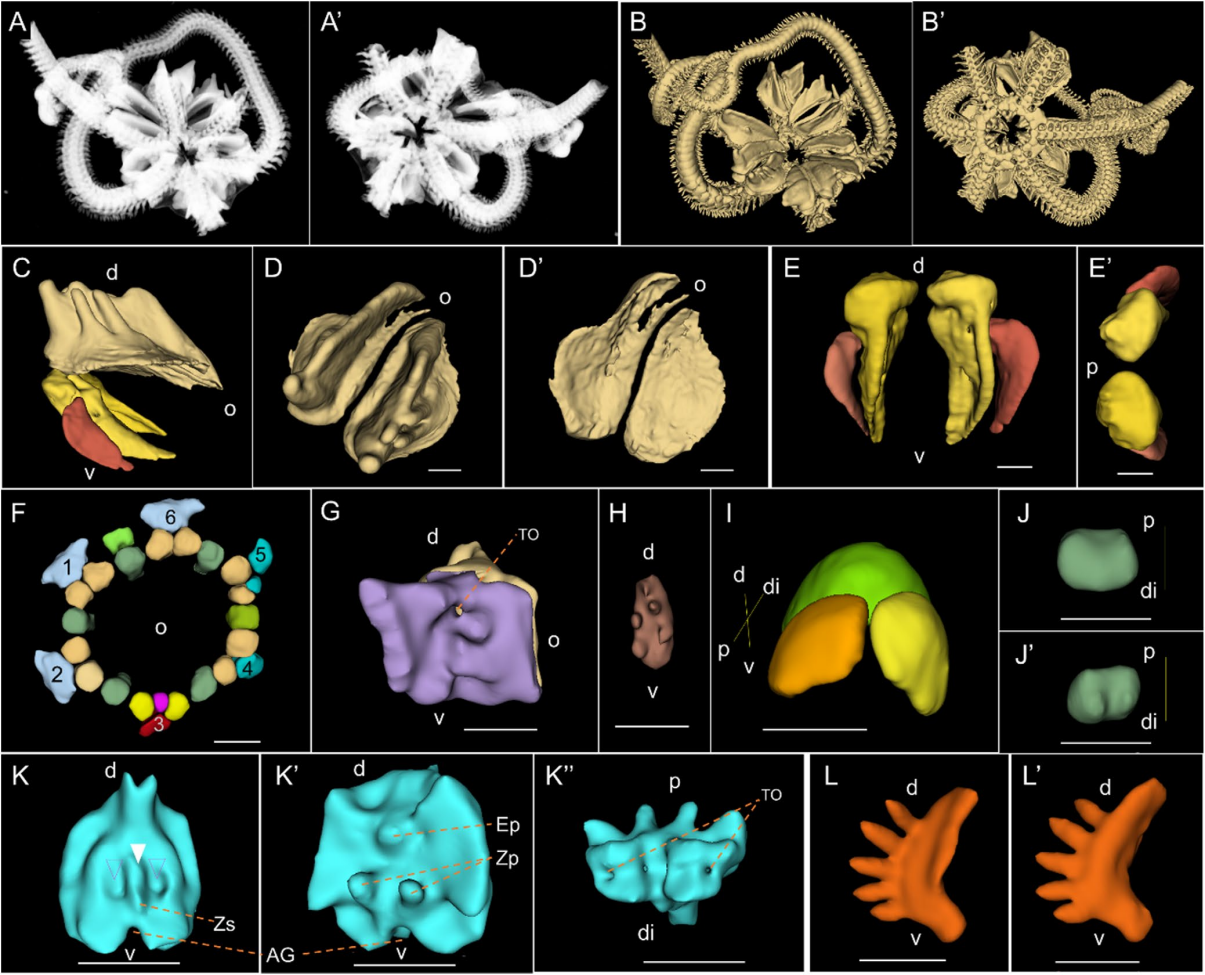
Micro CT segmentation of skeleton. (A, A') dorsal and ventral view of 3D rendering of the whole body; (B, B′) dorsal and ventral view of surface rendering of the whole body; (C–L') surface renderings of body parts. (C) combined view of radial shields (gold), adradial genital plates (yellow), and abradial genital plates (indian red); (D, D′) dorsal and ventral view of radial shield pair; (E, E') ventral view and radial view of adradial genital plates (yellow) and abradial genital plates (indian red); (F) dorsal view of oral shields (blue), adoral shields (yellow) and first ventral arm plate (green); (G) lateral view of two half jaws; (H) tooth plate with tooth or papilla; (I) dorsal view of dorsal arm plate (green) and split dorsal arm plate (orange and yellow); (J) ventral and dorsal view of ventral arm plate; (K, K′, K″) distal, proximal and ventral view of vertebrae; Zs: Zygosphene; Zp: Zygopophysis; Ep: Epanapophysis; AG: Ambulacral groov; (L, L') proximal and distal view of lateral plate with five spines. Scale bar is 1 mm; d, dorsal; v, ventral; di, distal; p, proximal; o, oral(mouth); TO, tubefeet opening.

The overall morphology of nearly all ossicles is clearly visible in both volume‐rendering (Figure 2A,A') and surface‐rendering images (Figure 2B,B'). The surface‐rendering images provide greater detail of each ossicle compared to the volume‐rendering images (Figure 2B,B'). The radial shields are concave on the ventral side, cover most of the dorsal disc but do not extend to the center, and also cover the first eight vertebrae. The crest of each radial shield swells near the outer edge of the disc (Figure 2C,D,D'). The outer edge of the radial shield articulates with the adradial genital plate, which is bar‐like with bulbous distal end, curved with dorsal edge concave, ventral edge convex, gradually tapering toward the oral side, similar to that of *Ophiothrix* (Stöhr 2024). The adradial genital plate articulates with the smaller, sickle‐shaped abradial genital plate, forming the genital slit (Figure 2C,E,E'). Both sides of the adradial genital plate contain multiple openings and grooves, likely for muscle attachment (Figure 2E,E'). A pair of curved‐bar‐like oral genital plates is present next to each oral shield. There are three large, continuous, diamond‐shaped oral shields and three smaller, continuous, irregular pentagonal oral shields, each adjacent to two pentagonal adoral shields. Six pairs of adoral shields and six first ventral arm plates form a ring around the mouth, with one thickened and partially hollow oral shield (i.e., the madreporite, dark red ossicle in Figure 2F, Figure S1). A small oval plate, positioned between the two adoral shields, connects to the madreporite (Figure 2F), and the fifth oral shield (OS5) is broken into two small pieces (Figure 2F). Among these ventral plates, the average stereom density of the oral shields is the highest (CT = 3415, *ρ* = 1.503 g/cm^3^), followed by the adoral shields (CT = 3321, *ρ* = 1.487 g/cm^3^), and the first ventral arm plates, which have the lowest density (CT = 3264, *ρ* = 1.476 g/cm^3^). The madreporite has the second‐highest stereom density (CT = 3540, *ρ* = 1.525 g/cm^3^), just below the fourth oral shield (OS4) (Figure 2F), while the small oval plate connecting the madreporite has the lowest density (CT = 2924, *ρ* = 1.417 g/cm^3^). The half‐jaw is triangular‐prismatic, located above the adoral shields, with tube foot openings visible extending from dorsal to lateral (Figure 2G). The stereom density of the two half‐jaws is lower (CT = 3059 and 3090, *ρ* = 1.441 g/cm^3^ and 1.446 g/cm^3^, respectively) compared with the oral and adoral shields (except the adoral shields next to the madreporite) and first ventral arm plates. All stereom densities of the above ossicles are supplied in Table S4. A dental plate with five teeth is attached to the tip of each pair of half‐jaws (Figure 2H).

Each arm segment consists of a central vertebra, surrounded by one or two dorsal arm plates, a ventral arm plate, and a pair of lateral arm plates bearing spines (Figure 2, Figure S2). Large conical protrusions and multiple irregular small ossicles are present on the dorsal side of the arm segment near the outer edge of the disc. There are two types of dorsal arm plates: one formed by a single complete rectangular to spindle‐shaped ossicle, and the other composed of a pair of oval ossicles (Figure 2I). Except for the first ventral arm plate, the ventral arm plates are rounded rectangular, with a thickness of about one‐third to one‐half of their width. The ventral arm plates and the V‐shaped depression on the ventral side of vertebrae (Figure 2J,J') form a groove (including the radial nerve and water canal) on the ventral side of each arm. The first ventral arm plate thickens toward the mouth, inserts between the outer edges of the two pairs of jaws, and is triangular in the lateral view. The dorsal surface of the vertebra is keeled distally, bifurcating at the top to form the radial water canal, in conjunction with the dorsal arm plate. A middle protrusion (Figure 2K, closed arrowhead) is present on the distal surface, with a joint socket (Figure 2K, open arrowhead) on both sides, forming the articulation with the tips of the epanapophyses and zygapophyses on the proximal surface (Figure 2K,K'). The zygocondyles on the distal surface are missing in this species (Figure 2K, Figure S2). The type of vertebral articulation and the large gap between neighboring ventral plates suggest that the horizontal movement range is greater than the vertical range, which likely enhances its ability to cling to the host crinoid (sea lily). Openings on the ventral side of the vertebra reveal obvious nerve and water canals belonging to the tube feet. The lateral arm plates are sickle‐shaped, with the ventral end extending into the space between adjacent ventral arm plates, leaving a large opening for the tube feet. No tentacle scales cover the opening. Most lateral arm plates possess five spines, with the three middle spines being stronger than the other two.

We compare our specimen with the *Gymnolophus* holotype in Brock (1888) (see Table S1 for more detailed comparison) and other publications (Nigam and Raghunathan 2016; Mekhova et al. 2018; Li et al. 2023). The shapes of the disc and arms are similar to those specimens except that our specimen is hexagonal and has six arms. The key feature is the large body size and large radial shield with high crest on it, which classifies it as *Gymnolophus* (Brock 1888). Other features like the shape of arm plates and number of spines are similar to *Gymnolophus obscura*. However, the adoral shields are different in shape and there is only a tiny space between radial shields without other plates or scales. Therefore, we named it *Gymnolophus sexradia* sp. nov. to indicate its major feature: hexamerous and six arms.

### 3.3 Characteristics of Mitogenome

**FIGURE 3:**
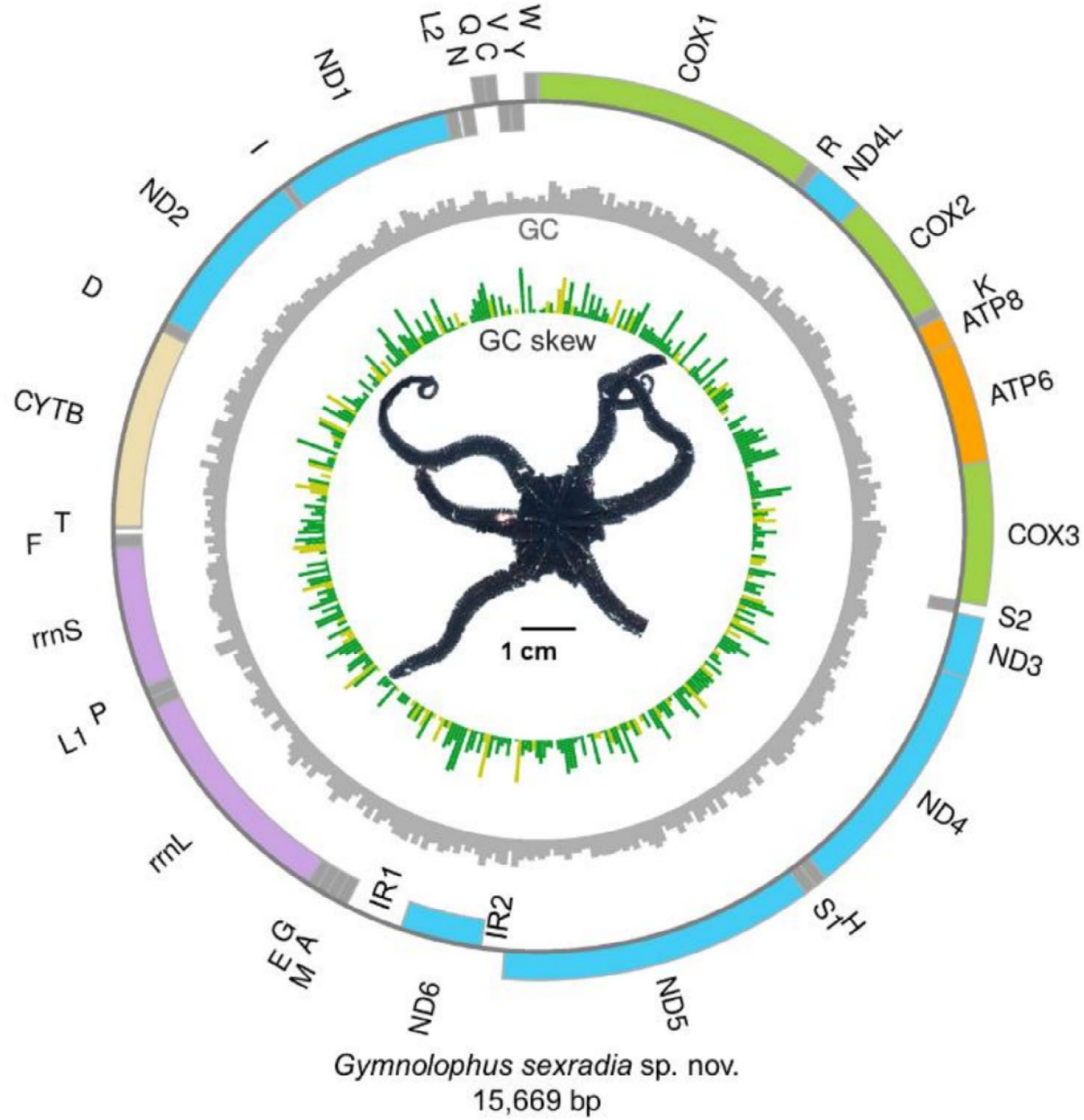
Circular map of sequenced mitogenome of *Gymnolophus sexradia* sp. nov. circular tracks from inside to outside is GC skew, GC percentage with window size 40 bp and genes. Yellow bars in the GC skew track indicated positive value and green ones were negative value. Outward (inward) genes are on the heavy (light) strand.

The complete mitochondrial (MT) genome of *Gymnolophus sexradia* sp. nov. has a total length of 15,669 bp. The base composition consists of 34.9% A, 30.2% T, 20.2% C, and 14.7% G, with a GC content of 34.9%. The genome contains 37 genes and two intergenic regions (IRs) flanking ND6, with sizes of 139 bp (IR1) and 347 bp (IR2), respectively (Figure 3). Each IR contains a replication origin. The GC‐skew of the entire MT genome is −0.155, which is similar to that of *Macrophiothrix demessa* (−0.157) and *Ophiothrix* spp. (−0.175 to −0.148) (Table S2). The genome includes 13 typical protein‐coding genes (COIII, ND1‐6, ND4L, ATP6, ATP8, and CYTB), 2 rRNA genes (16S‐rRNA [rrnL], 12S‐rRNA [rrnS]), and 22 tRNA genes. Among the 13 protein‐coding genes, only COI and ND1 use GTG as the start codon, while all other genes use ATG as the start codon. The stop codons for CYTB, ND4, and ND1 are TAG, while the remaining genes terminate with TAA.

**FIGURE 4:**
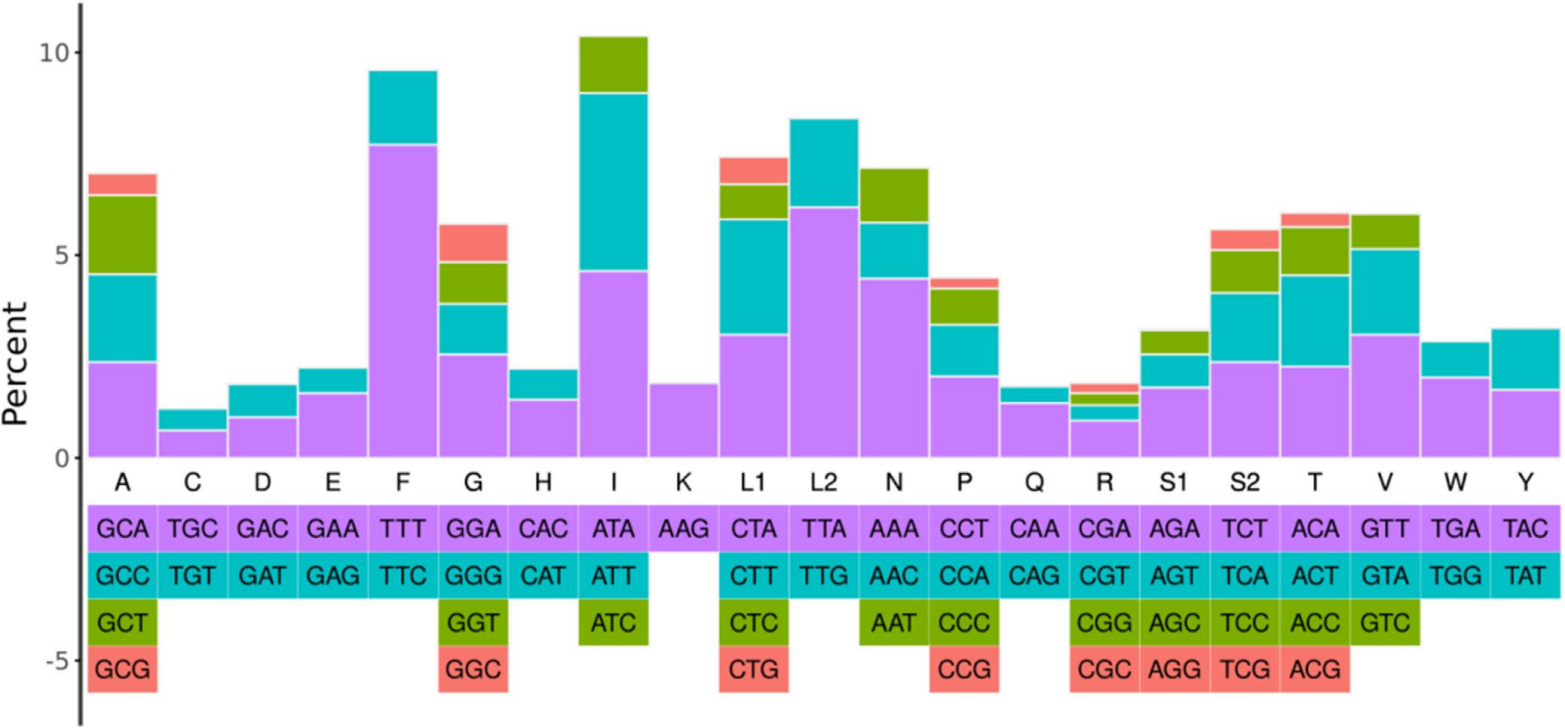
Relative synonymous codon usage (RSCU) in *Gymnolophus sexradia* sp. nov. Abbreviations for codon families are labeled on the x‐axis.

**FIGURE 5:**
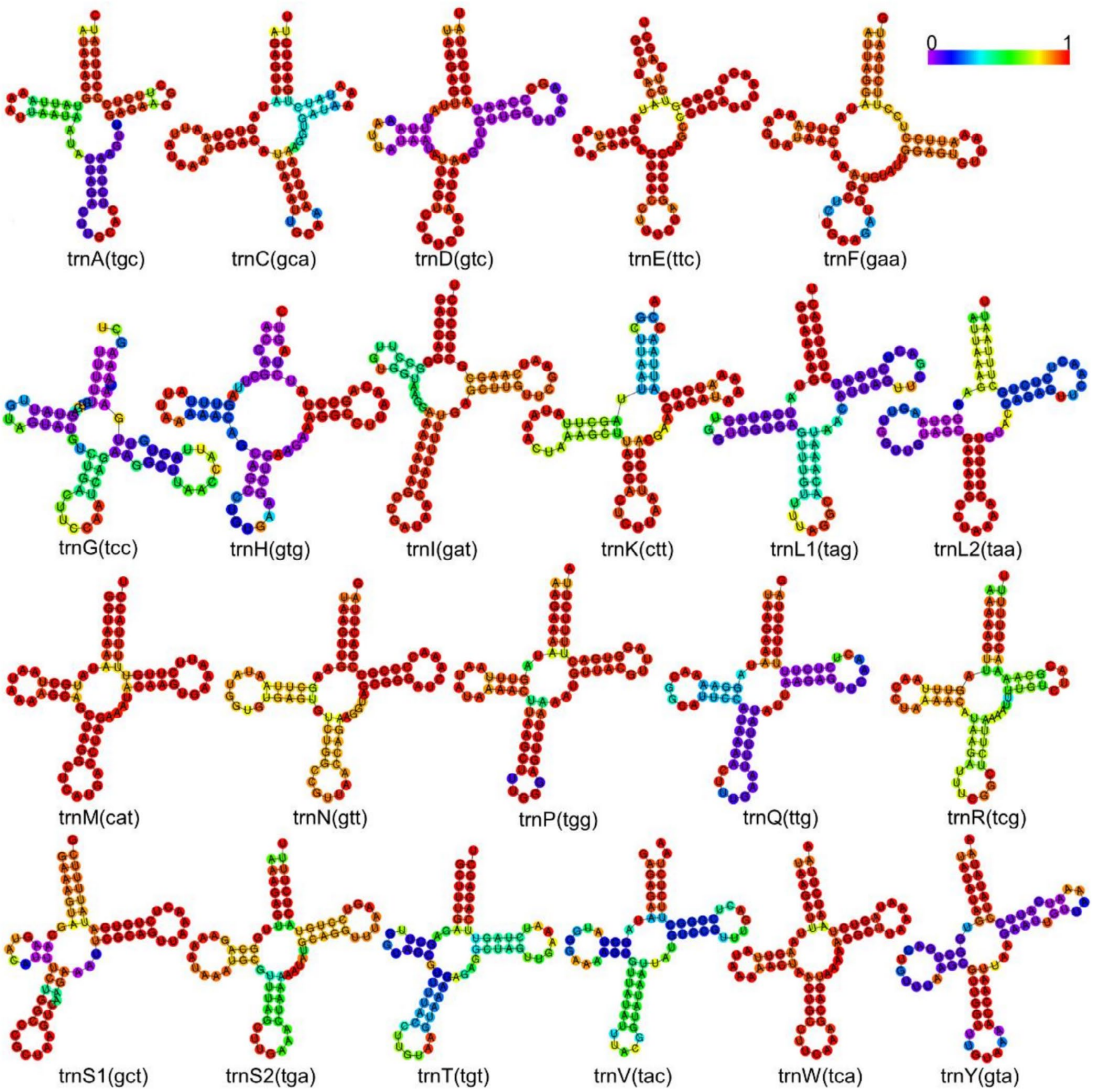
Secondary structures of the 22 tRNA, base pairs are colored by base‐pair probabilities.

The ATP8 gene has the highest A + T content at 77.59%, whereas the COIII gene has the lowest A + T content at 58.27%. Analysis of the amino acid composition and corresponding synonymous codons revealed that isoleucine has the highest frequency of codon usage, while cysteine has the lowest frequency. Among the 22 tRNA genes, the most frequently used anti‐codon is TTT (for phenylalanine, Phe), while the least frequently used anti‐codons are CGC (for arginine, Arg) and CCG (for proline, Pro) (Figure 4). The sizes of the 22 tRNA genes range from 66 bp to 75 bp. All tRNAs could form a clover‐leaf secondary structure (Figure 5), with the minimum free energy ranging from −28.82 (trnW) to −9.91 (trnR). All structures exhibit discrimination bases in the acceptor stem, with mismatches observed in the acceptor stem of trnE, trnH, trnK, trnV, and the T‐arm of trnG and trnY.

### 3.4 Phylogenetic Analysis

**FIGURE 6:**
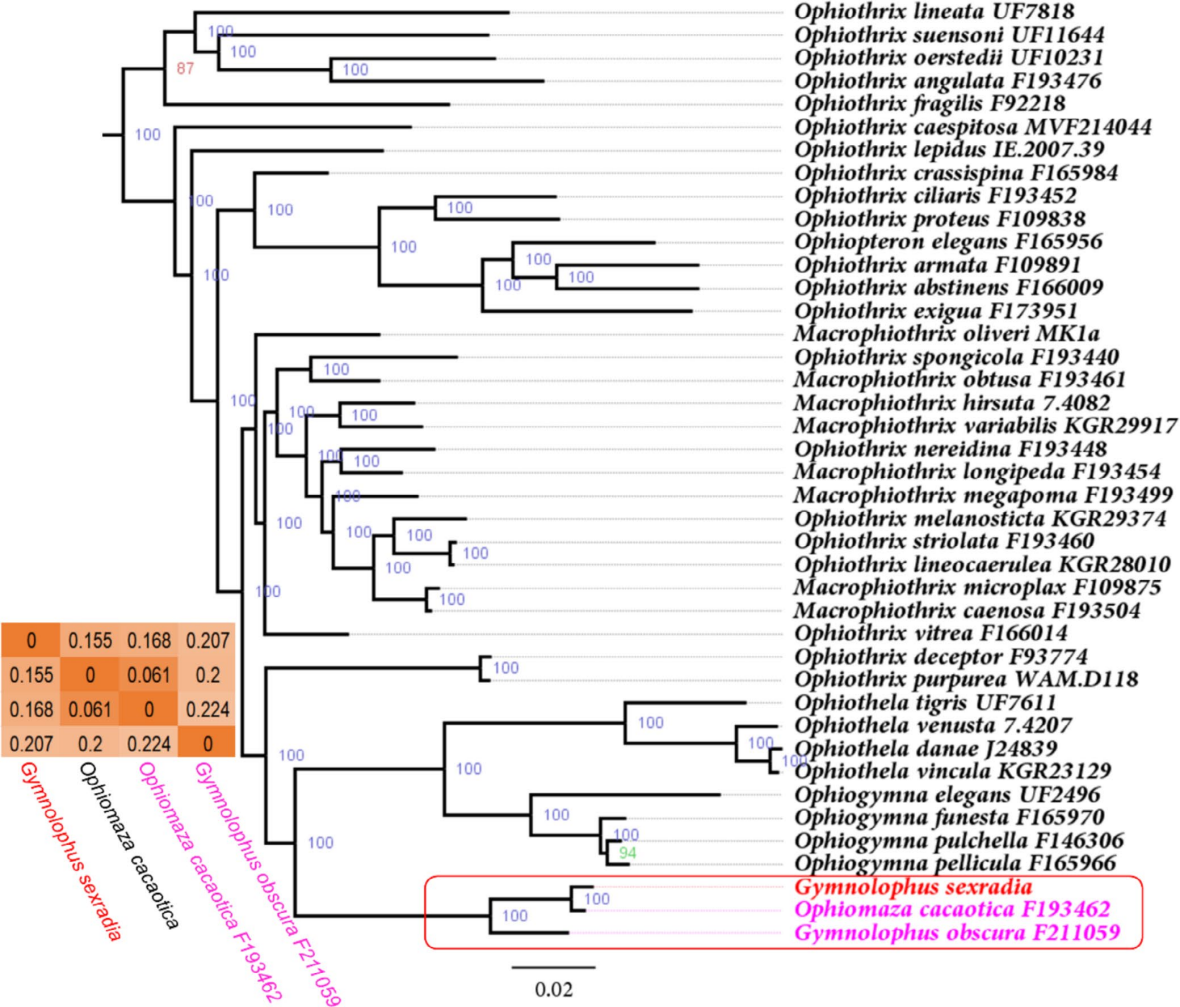
Phylogeny of Ophiotrichidae based on 1464 exons from (O'Hara et al. 2017). *Gymnolophus sexradia* sp. nov. (red taxon name) clades with *Ophiomaza cacaotica* F193462 and is placed between *Gymnolophus obscura* F211059 (magenta taxon name) and *Ophiomaza cacaotica* F193462 (magenta taxon name). Number on nodes indicate bootstrap values, colored by values. Bottom left is kimura distance of COI nucleotide sequences between *Gymnolophus sexradia* sp. nov. and *Gymnolophus obscura* F211059, *Ophiomaza cacaotica* (include F193462) in the red box.

**FIGURE 7:**
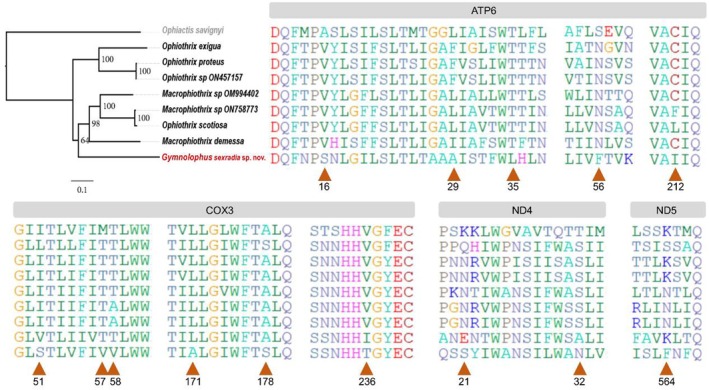
Phylogenetic tree of nine brittle star species was constructed using the Maximum Likelihood Method based on amino acid sequences, with *Ophiactis savignyi* (NC_065087) as the outgroup (color gray). Branch length indicates substitution per site. Triangles and numbers under sequences indicate position of positive selective sites.

The phylogenetic analysis of the exon sequences within Ophiotrichidae indicates our specimen is clustered with *Ophiomaza cacaotica* (isolate F193462) and placed between it and *Gymnolophus obscura* (isolate F211059) (Figure 6). The Kimura distance of COI sequences between our specimen and *O. cacaotica* (or *G. obscura* , for which the distance is slightly larger) exceeds 0.2, indicating that our specimen is a new species different from them (Figure 6). The epizoic lifestyle may exert selective pressure on mitochondrial genes. Using the branch‐site model, a signal of positive selection was detected in COIII (*p* = 0.058), and a weaker signal was observed in ND4 (*p* = 0.083). Additionally, 14 residues were identified as positively selected sites with high or medium posterior probabilities (Bayes empirical Bayes (BEB) values > 0.9) in ATP6 (5 sites), COIII (6 sites), ND4 (2 sites), and ND5 (1 site) (Figure 7).

The relative conservation of the mitochondrial genome within the family Ophiotrichidae was revealed through comparative analysis of the gene order in *Gymnolophus sexradia* sp. nov. and eight other species (Figure S3). The gene order for the 13 protein‐coding genes (PCGs), 2 rRNA genes, and 22 tRNA genes was conserved within Ophiotrichidae, as well as between Ophiotrichidae and *Ophiactis savignyi* .

## 4 Discussion

### 4.1 Taxonomic Implications

Morphologically, Adults of *G. obscura* typically possess a pentamerous body plan with five arms, characterized by a naked disc and well‐developed longitudinal crests on the radial shields and split dorsal arm plates on some arm segments. The specimen in this study shares these morphological traits, but differs in its hexamerous adult body plan (with six arms) and distinct morphology of adoral shields (Brock 1888). Phylogenetic analysis of exons with Ophiotrichidae places our specimen between *G. obscura* and *Ophiomaza cacaotica* , and the COI Kimura distances between our specimen and *O. cacaotica* or *G. obscura* are relatively large, indicating that our specimen represents a new species distinct from them. Therefore, we identify it as *Gymnolophus sexradia* sp. nov. Hexamerous ophiuroids are usually (but not always) fissiparous, and show half smaller disc, three larger and three smaller arms during asexual reproduction (Stöhr 2003). The hexamerous body, small oral shields and some corresponding adoral shields on one side of the disc of our specimen may indicate it is fissiparous. Additionally, we collected a pair of specimens from the same crinoid, both hexamerous. Future studies integrating population genetics and broader sampling are needed to clarify the true nature of this six‐armed morphology, whether it is fissiparous, and to further resolve the phylogenetic relationships and diagnostic boundaries between the genera *Gymnolophus* and *Ophiomaza*.

### 4.2 Morphological Adaptation to Symbiotic Lifestyle

The morphology of the arm vertebrae and the structure of their articulations directly constrain the bending and twisting capabilities of the arms, and these features are closely related to ecological habits (e.g., epizoic or endozoic) (Goharimanesh et al. 2022). There are two main types of vertebral articulations: streptospondylous vs. zygospondylous articulation (Goharimanesh et al. 2022). The vertebral articulation of *G. sexradia* is a reduced zygospondylous articulation. The epanapophyses on the distal side of the vertebrae are normal in shape, and the gap between adjacent dorsal arm plates is small, which restricts dorsal (upward) bending of the arms. However, the zygosphene is shortened, and the zygocondyles are lost on the distal side of the vertebra. The reduced zygosphene and lack of zygocondyles, combined with the large space existing between adjacent ventral arm plates, provides this brittle star with considerable horizontal (lateral) flexibility. Middle segments of dorsal arm plates also possess more split dorsal arm plates, which overlap each other to increase the horizontal (lateral) flexibility with curvature larger than 1/2.7 mm^−1^ (Figure S4). This enhanced horizontal flexibility facilitates curling the arms and tightly grasping the calyx or arms of its crinoid host while still keeping its mouth facing crinoid mouth for feeding, which is considered one of the key morphological adaptations of epizoic brittle stars (Stöhr 2025). Furthermore, *G. obscura* is known to migrate between different individual crinoids, a process during which the brittle star may be exposed to predators; thus, rapid arm grasping and coiling ability provides a survival advantage (Mekhova et al. 2018).

### 4.3 Mitogenome Characteristics and Evolution

The complete mitochondrial genome of *G. sexradia* is 15,669 bp in length and contains 37 genes. Its base composition shows a high A + T content, which is consistent with the mitochondrial genome characteristics of other known species in the family Ophiotrichidae. A high A + T content is commonly found in other nonepizoic echinoderms and is not unique to epizoic species (Galaska et al. 2019; Li et al. 2021; Na et al. 2019). Analysis of mitochondrial gene order indicates that the gene order of *G. sexradia* is completely identical to that of the congeneric *Macrophiothrix* and *Ophiothrix*, and also the same as that of the outgroup *Ophiactis savignyi* . This gene order is considered typical of the ancestral superorder Ophintegrida and differs from that of species in the superorder Euryophiurida (Galaska et al. 2019; Sun et al. 2023). The high conservation of mitochondrial gene order may reflect evolutionary constraints on mtDNA transcriptional patterns (Shtolz and Mishmar 2023) rather than a direct adaptation to a symbiotic lifestyle. Overall, the mitochondrial genome of *G. sexradia* sp. nov. conforms to the general pattern of the class Ophiuroidea in terms of composition and structure, with no specialized rearrangements or extreme base composition bias significantly associated with its epizoic habit.

### 4.4 Molecular Signatures of Adaptation

Different lifestyles can influence the evolution of mitochondrial genomes; for example, the mitochondrial genomes of chemosynthetic deep‐sea mussels can be used to explore adaptation mechanisms in deep‐sea symbiotic taxa, and differences between parasitic and free‐living lifestyles may also affect the evolutionary rates of mitochondrial genomes in bilaterians (Jakovlic et al. 2023; Zhang et al. 2021). positive selection signals were detected in the mitochondrial protein‐coding genes ATP6, COIII, ND4, and ND5 in new species which may be related to mitochondrial functional variation and energy metabolism (Awadi et al. 2021; Li et al. 2018). It is speculated that the detected positive selection signals may be related to metabolic demands associated with an epizoic lifestyle. Because epizoic brittle stars living on crinoids need to continuously attach to their host, which may increase energy expenditure in the tube feet and arm muscles (Nigam and Raghunathan 2016; Li et al. 2023). Meanwhile, research on other marine invertebrate symbiotic systems reveals that symbiotic relationships can alter the host's nutrient acquisition and metabolic balance. For instance, nutrient availability and metabolic processes can influence the stability of the coral‐symbiotic algal relationship. The coupled carbon and nitrogen cycles regulate the nutrient exchange between cnidarians and algae, while chemosynthetic symbionts provide organic carbon and other metabolic resources for marine invertebrate hosts (Morris et al. 2019; Rädecker et al. 2023; Sogin et al. 2020). However, the functional significance of these positively selected sites still requires further confirmation through comparative studies with closely related non‐epizoic taxa and experimental validation.

## 5 Conclusions

We described a new specimen collected from the South China Sea, diagnosed as similar to *Gymnolophus obscura*, and named *Gymnolophus sexradia* sp. nov. to highlight its hexamerous body plan. We described morphological characters and the density of its ossicles using external observation and micro‐CT, including internal ones like vertebrae and genital plates. We also provided its complete mitogenome sequence, which is 15,669 bp in length and contains all 37 typical mitochondrial genes. The gene order is conserved among the genera *Gymnolophus*, *Macrophiothrix*, and *Ophiothrix* within the family Ophiotrichidae. Additionally, 14 positively selected residues were identified across four mitochondrial coding genes. The exon phylogenetic analysis within Ophiotrichidae also places the new species between *G. obscura* and *O. cacaotica* . The vertebral articular structure and the positively selected sites likely reflect adaptations to its epizoic life with host crinoids. Further sampling and genetic analysis are needed to clarify whether it is fissiparous and its relationship with host species.

## Author Contributions

**Ziye Zhang:** conceptualization (equal), data curation (equal), investigation (equal), resources (equal), writing – original draft (equal), writing – review and editing (equal). **Weilin Xiao:** data curation (equal), resources (equal), software (equal), supervision (equal), visualization (equal). **Chao Zhang:** writing – review and editing (equal). **Chunyan Li:** writing – review and editing (equal). **Huixian Zhang:** writing – review and editing (equal). **Zelin Chen:** funding acquisition (equal), supervision (equal), validation (equal), writing – original draft (equal), writing – review and editing (equal).

## Funding

This work was supported by the National Key Research and Development Program of China, 2021YFF0502803 and 2023YFC2811501, National Natural Science Foundation of China, 42276135, GuangDong Basic and Applied Basic Research Foundation, 2024A1515010348, National Science Foundation of China, 32573511, and Guangdong Youth Talent Program, 2021QN02H979.

## Conflicts of Interest

The authors declare no conflicts of interest.

## Supporting information


**Figure S1:** Paired curved‐bar‐like oral genital plates adjacent to each oral shield; Channel (dark inside, low CT values) in oral shields (OS2) and (OS3, the madreporite) in volume rendering.

**Figure S2:** Observation of arm plates under microscope. Yellow scaler is 1 mm. Yellow arrow heads indicate articular structure on lateral arm plate. One lateral side of the vertebrate is broken at bleaching.

**Figure S3:** Gene order of the mitochondrial genome of *Gymnolophus sexradia* sp. nov with the other eight species.

**Figure S4:** The bifurcated structure in the middle of the dorsal arm plate and its lateral flexibility; A middle segment of arms of *Gymnolophus sexradia* sp. nov. after bleach. Dosal view. Red lines show the curvature radius of the segment.

**Table S1:** This specimen is compared with the morphological characteristics of the type specimen of *Gymnolophus* described by Brock (1888) and other specimens documented in literature.

**Table S2:** Information on 9 mitochondrial genomes of Ophiotrichidae for evolutionary analysis.

**Table S3:** Kimura distance of the COI gene for species in the Ophiotrichidae.

**Table S4:** Micro‐CT data of the ossicles of *Gymnolophus sexradia* sp. nov.

**Video S1:** 3D animation of the complete skeletal structure of *Gymnolophus sexradia* sp. nov. from micro‐CT data.

**Video S2:** 3D animation of the major arm ossicles of *Gymnolophus sexradia* sp. nov. from micro‐CT data.

## Data Availability

The complete mitochondrial genome sequence generated in this study has been deposited in Dryad. A private, temporary link is provided for peer review purposes: https://doi.org/10.5061/dryad.rxwdbrvr4. Upon acceptance of the manuscript, the dataset will be made publicly available with a permanent DOI.
