# Supplementary figures and images for: Morphological Characterization and Complete Mitochondrial Genome of a New Epizoic Hexamerous Brittle Star Gymnolophus sexradia sp. Nov
Source: Ecol Evol. 2026 Jul 28;16(8):e73841. doi: 10.1002/ece3.73841 (PMC13416197; doi:10.1002/ece3.73841)

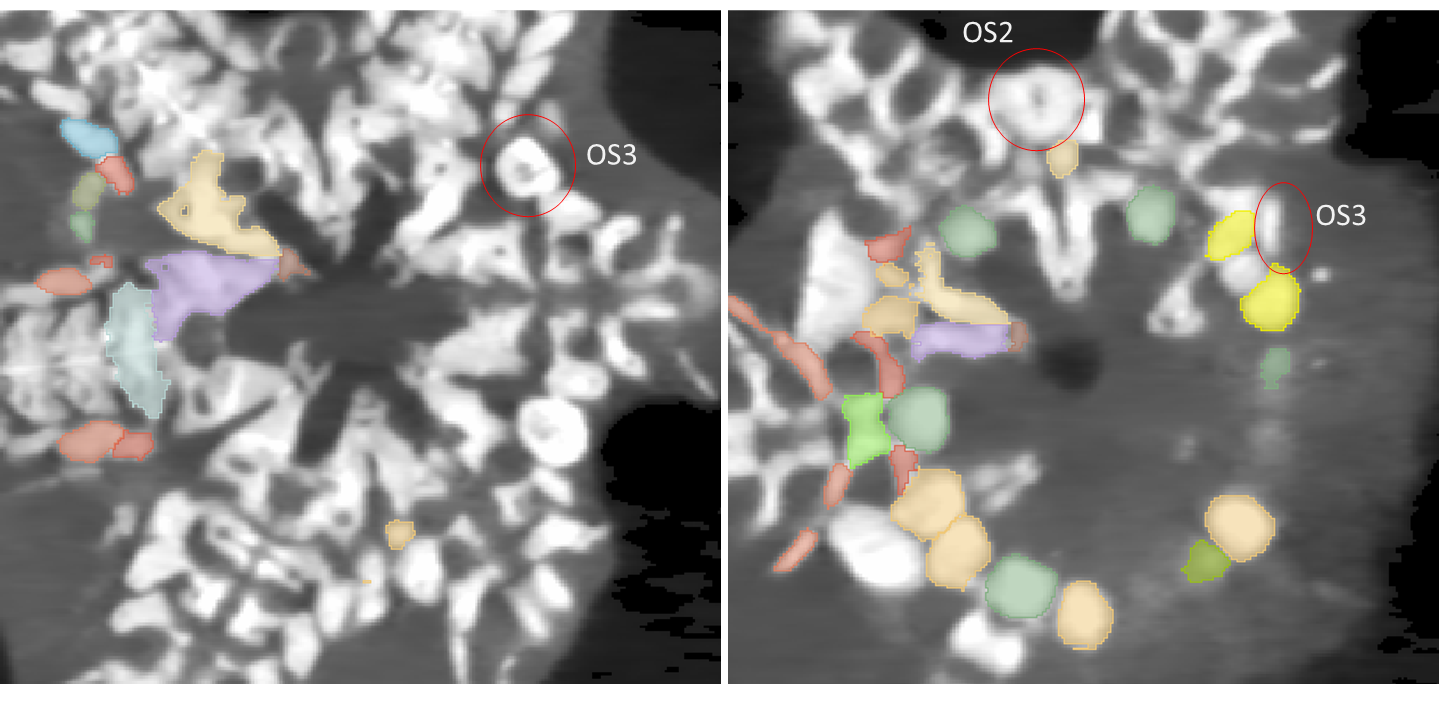

Supplement: Supplementary file 1 — Figure S1: Paired curved‐bar‐like oral genital plates adjacent to each oral shield; Channel (dark inside, low CT values) in oral shields (OS2) and (OS3, the madreporite) in volume rendering. [file ECE3-16-e73841-s009.tiff]

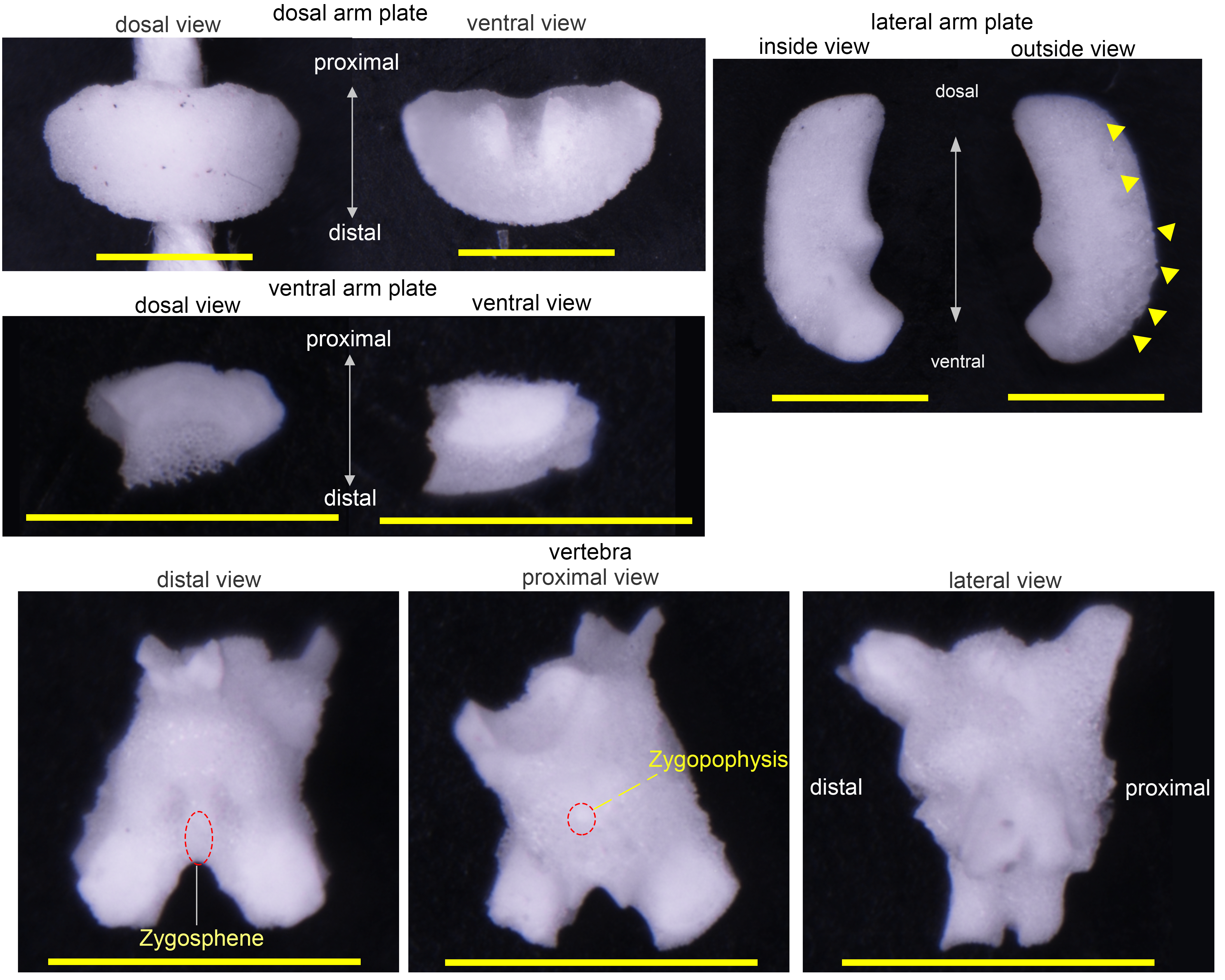

Supplement: Supplementary file 2 — Figure S2: Observation of arm plates under microscope. Yellow scaler is 1 mm. Yellow arrow heads indicate articular structure on lateral arm plate. One lateral side of the vertebrate is broken at bleaching. [file ECE3-16-e73841-s002.tif]

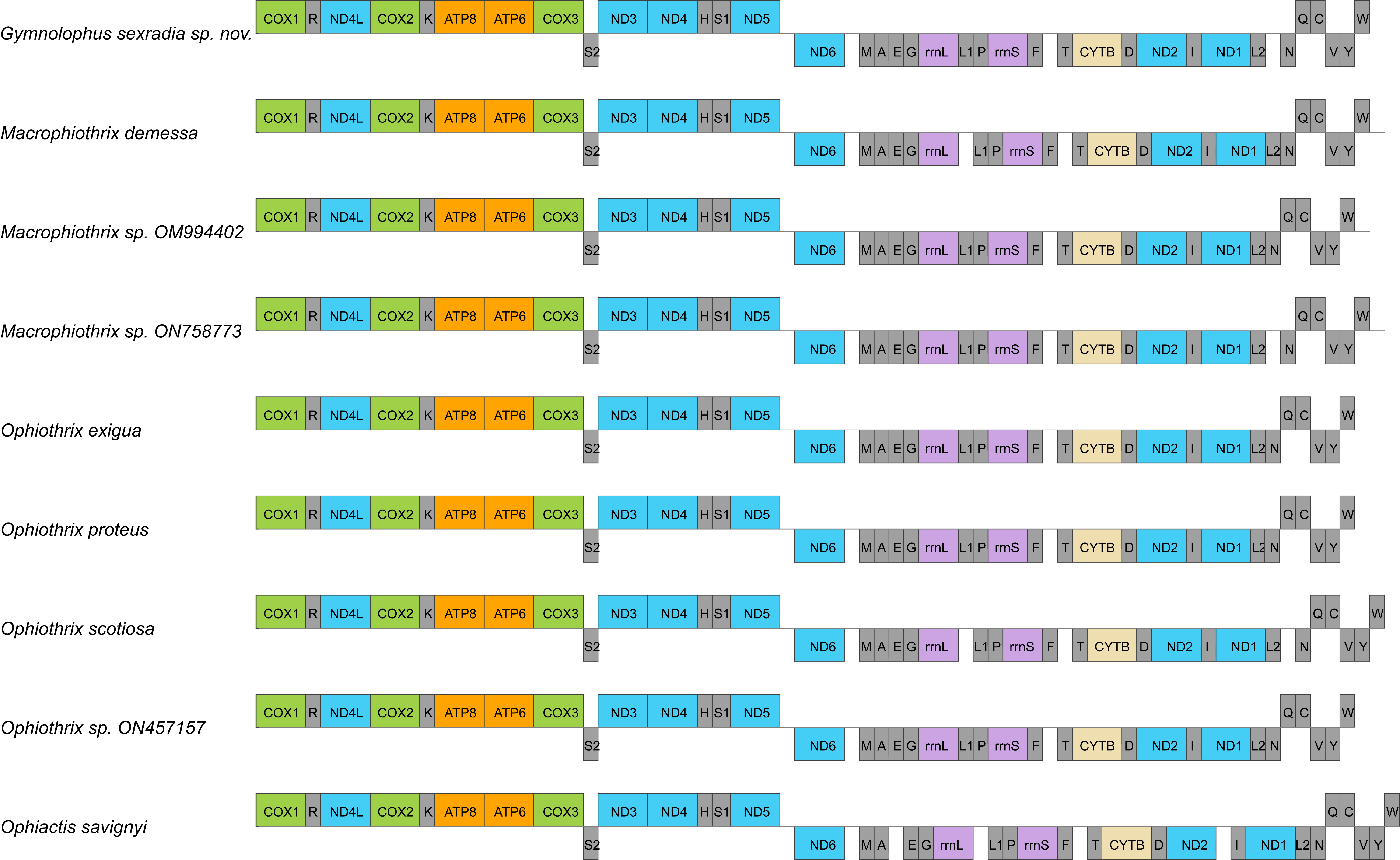

Supplement: Supplementary file 3 — Figure S3: Gene order of the mitochondrial genome of Gymnolophus sexradia sp. nov with the other eight species. [file ECE3-16-e73841-s004.tif]

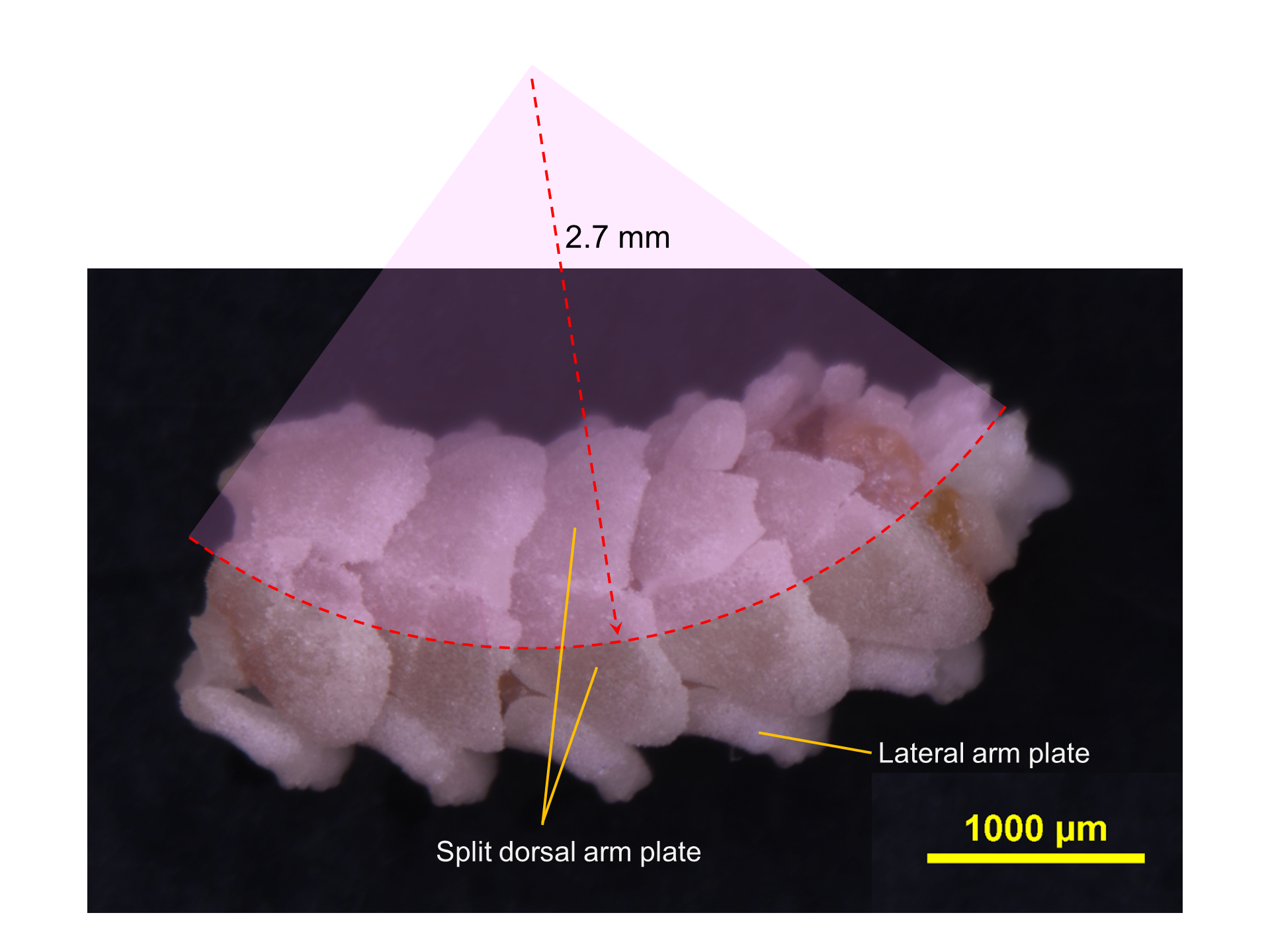

Supplement: Supplementary file 4 — Figure S4: The bifurcated structure in the middle of the dorsal arm plate and its lateral flexibility; A middle segment of arms of Gymnolophus sexradia sp. nov. after bleach. Dosal view. Red lines show the curvature radius of the segment. [file ECE3-16-e73841-s003.tif]
